# Parental Alcohol Use Disrupts Offspring Mitochondrial Activity, Promoting Susceptibility to Toxicant-Induced Liver Cancer

**DOI:** 10.14336/AD.2024.1372

**Published:** 2026-01-25

**Authors:** Alison Basel, Sanat S. Bhadsavle, Katherine Z. Scaturro, Grace K. Parkey, Yava Jones-Hall, Michael C. Golding

**Affiliations:** ^1^Department of Veterinary Physiology & Pharmacology, College of Veterinary Medicine and Biomedical Sciences, Texas A&M University, College Station, TX 77843, USA.; ^2^Department of Veterinary Pathobiology, College of Veterinary Medicine and Biomedical Sciences, Texas A&M University, College Station, TX 77843, USA.

**Keywords:** paternal effects, mitochondrial activity, oxidative stress, hepatocellular carcinoma, epigenetic inheritance of cancer, Paternal Origins of Health And Disease (POHAD)

## Abstract

The early onset and incidence of liver disease and hepatocellular carcinoma have doubled in the last two decades and are primarily attributed to an unhealthy lifestyle. However, emerging studies suggest that increases in these age-related pathologies may link to heritable alterations in the control of cellular bioenergetics induced by the parental environment. Because our preclinical studies examining the fetal offspring of alcohol-exposed males and females have consistently identified epigenetic alterations in mitochondrial activity, we hypothesized that chronic parental alcohol exposure programs an increased predisposition of offspring to develop liver disease and hepatocellular carcinoma induced by an environmental toxicant. Here, we employed a multiplex mouse model to compare the sensitivities of male offspring derived from maternal, paternal, and dual-parental alcohol exposures to the potent hepatocellular carcinoma inducer Diethylnitrosamine and determine their predisposition for tumor formation and growth. Our analysis reveals that parental alcohol exposures disrupt the activity of offspring mitochondrial complex I in the liver, promoting enduring oxidative stress and activating Transforming Growth Factor β signaling. This lasting imbalance correlates with increased Interleukin 6 production, promoting an inflammatory precancerous state. In male offspring, chronic parental alcohol consumption leads to increased tumor incidence, multiplicity, and size. Significantly, maternal and paternal alcohol use interact in driving the progression of toxicant-induced liver disease, with some adverse outcomes of dual-parental offspring exceeding those caused by either maternal or paternal alcohol use alone. We conclude that chronic parental alcohol use alters mitochondrial complex I activity and immune function, predisposing male offspring to a proinflammatory precancerous state.

## INTRODUCTION

There is a growing interest in understanding how lifestyle choices and social factors affect healthspan, life expectancy, and the onset of chronic age-related diseases, including cancer [1]. In addition to direct exposures, researchers now suspect that stressors encountered by an individual’s parents also shape the determinants of life-long health, particularly stressors experienced during the periconceptional period, which may influence the establishment of the embryonic epigenome and inherent aspects of disease susceptibility [2, 3].

Although the importance of early life stressors and maternal lifestyle choices during pregnancy have been well characterized, paternal contributions to healthspan and age-related disease are comparatively unknown and often not considered [4, 5]. Nonetheless, recent studies examining paternal preconception lifestyle choices and exposure histories demonstrate that environmentally-induced alterations in male germline epigenetic programming also influence offspring behavior, growth, and metabolic health [4, 6]. However, researchers have yet to determine whether preconception male exposures directly contribute to the incidence of chronic disease or if maternal and paternal exposures interact in predisposing offspring to adverse health outcomes.

Cancer is now understood to have both a genetic and environmental component, with epigenetic processes bridging the gap between environmental stressors and genetic predispositions [7, 8]. Although the role of epigenetic changes or ‘epimutations’ in cancer progression is still emerging, the prevailing assumption in this area of research is that cancer-causing epigenetic changes occur during the affected individual's lifetime. In contrast, the potential for environmentally-induced epigenetic changes in the parental germline to heritably influence cancer development and progression in offspring is only just emerging and has not been thoroughly investigated [9].

Alcohol is a known carcinogen that accounts for roughly one-third of primary liver cancer cases worldwide [10, 11]. However, it is not yet known if chronic alcohol consumption by an individual’s parents heritably influences their predisposition to develop liver cancer. Clinical studies demonstrate that children with fetal alcohol syndrome exhibit an increased incidence of liver disease, including hepatic fibrosis, cirrhosis, and metabolic abnormalities [12-16]. Likewise, maternal alcohol consumption during pregnancy is associated with an increased risk of developing leukemia, and preclinical studies in rodents demonstrate that gestational alcohol exposures enhance offspring predisposition to tumorigenesis [17-22]. However, whether these adverse effects result from direct alcohol exposures, ethanol is mutagenic *via* its first metabolite, acetaldehyde, or arises through heritable epigenetic changes in gene expression remain unknown.

Metabolic dysfunction-associated steatotic liver disease (MASLD), formerly referred to as non-alcoholic fatty liver disease (NAFLD), is the leading primary cause of chronic liver disease in both children and adults [23]. The global prevalence and early onset of MASLD are rapidly rising, particularly among young men [24]. Indeed, over the past 30 years, MASLD prevalence has increased from 25% to 35%, and it is now estimated to affect nearly one-third of individuals in the United States [23-25]. MASLD is a progressive disorder beginning with simple steatosis and advancing to more serious non-alcoholic steatohepatitis (NASH), which occurs in the absence of excessive alcohol intake [26]. Subsequent macrophage infiltration and inflammation lead to irreversible fibrosis and cirrhosis, which, if left untreated, can progress to hepatocellular carcinoma (HCC), the last stage of MASLD [27]. Although the rising incidence of MASLD and NASH in young males is primarily attributed to the unhealthy lifestyle choices of affected individuals, emerging preclinical studies suggest some aspects of disease progression may be linked to environmentally induced changes in the epigenetic regulation of cellular bioenergetics inherited from their parents [28].

In our preclinical studies examining the fetal offspring of alcohol-exposed males, we have consistently observed adverse paternal influences on the transcriptional regulation of mitochondrial function [29-32]. We and others find that the phenotypic and behavioral changes induced by paternal alcohol use correlate with alterations in sperm noncoding RNAs, many of which converge on the regulation of the nuclear factor erythroid 2-related factor 2 (Nrf2)-driven cellular antioxidant response and the transcriptional pathways controlling mitochondrial dynamics [32-34]. Similarly, we have described alterations in transcriptional regulation of mitochondrial function in a mouse model of maternal alcohol exposure [35], while using a dual-parental exposure model, we recently observed that the adult offspring of alcohol-exposed males and females exhibit increased markers of mitochondrial and oxidative stress, a decline in the cellular NAD+/NADH ratio, increased production of proinflammatory cytokines, and histological indicators of accelerated age-related liver disease [36]. Strikingly, in the male offspring, we observed an interaction between maternal and paternal alcohol use, with histological indicators of age-related liver disease in the dual-parental offspring exceeding those induced by either maternal or paternal alcohol use alone [36].

The observed increases in established markers of hepatic inflammation, liver disease, and compromised mitochondrial activity prompted us to consider the possibility that these lasting changes may increase offspring susceptibility to liver cancer. Indeed, compromised mitochondrial activity and an altered redox state drive HCC initiation and progression [37], and there is a growing interest in understanding the role of inflammation in accelerating biological aging and driving liver disease [38]. Moreover, increased proinflammatory cytokines, like Interleukin 6 (IL-6) observed in the offspring of alcohol-exposed parents [36], play a critical role in HCC development, proliferation, invasion, metastasis, and drug resistance [39, 40]. Given the emerging role of mitoepigenetics in driving chronic inflammation and modulating cancer progression, [41] we hypothesized that chronic parental alcohol exposure programs an increased predisposition of offspring to develop hepatocellular carcinoma induced by a "second hit" in the form of an environmental stressor.

Here, we employed our established multiplex mouse model [36, 42] to compare sensitivities of the male offspring of maternal, paternal, and dual-parental alcohol exposures to the potent hepatocellular carcinoma inducer, Diethylnitrosamine (DEN) [43]. The hepatic metabolism of the nitrosamine DEN by the cytochrome P450 enzyme CYP2E1 results in the production of alkylating metabolites, causing DNA adduct formation, severe liver fibrosis, and inflammation, and significant changes in liver mitochondrial function resulting in a reduced NAD+/NADH ratio and increased ROS production [44-47]. Collectively, these adverse effects promote hepatocarcinogenesis, making early-life DEN exposure a highly reproducible inducer of liver disease and hepatocellular carcinoma in mice [48]. Using this agent as a compounding secondary stressor, we repeated our previous dual-parental experiments and examined the ability of parental alcohol use to increase offspring predisposition to DEN-induced tumor formation and growth. Our experiments reveal that chronic parental alcohol consumption programs a pro-tumor environment in the liver. This increased cancer susceptibility correlates with increased steatosis, hepatic fibrosis, inflammation, oxidative stress, and compromised mitochondrial complex I activity. Interestingly, many of these changes display accumulative effects and are more pronounced when both parents are exposed to alcohol.

## MATERIALS AND METHODS

### Study Approval

We conducted all experiments following the procedures outlined under AUP IACUC 2023-0186, which was approved by the Texas A&M University IACUC. We performed all experiments following the guidelines and regulations of the Texas A&M IACUC and the National Research Council's Guide for the Care and Use of Laboratory Animals. Here, we report our data following the ARRIVE guidelines.

### Sex as a biological variable

Our previous studies reveal that paternal alcohol exposures consistently induce sex-specific changes in offspring fetoplacental growth and patterning, with male offspring exhibiting more severe outcomes than females [29, 30, 42, 49]. Similarly, previous studies in mice reveal that repeated DEN exposures induce hepatic tumors in 100% of exposed males, while only 30% of females develop hepatocellular carcinoma [43]. Finally, as the prevalence of liver disease is significantly higher among men than among women, [24] we focused our analyses on combined interactions between parental alcohol use and offspring DEN exposures in male offspring.

### Animal husbandry and alcohol treatments

We implemented the multiplex 2x2 dual-parental alcohol exposure model using previously published procedures [36, 42]. Briefly, we obtained adult C57BL/6J (Strain #:000664 RRID: IMSR_JAX:000664) mice from the Texas Institute of Genomic Medicine (TIGM) and maintained them in the TIGM facility on a reverse 12-hour light/dark cycle (lights off at 8:30 am) with *ad libitum* access to a standard breeder diet (catalog# 2019, Teklad Diets, Madison, WI, USA) and water.

On postnatal day 83, we individually caged male mice and allowed them to acclimate to individual housing for one week. As part of our animal husbandry, we introduced extra enrichments to the animal's home cage to mitigate the stress associated with individual housing. Specifically, we added shelter tubes to the males' home cages and provided igloos for the females (catalog# K3322 and catalog# K3570 from Bio-Serv, Flemington, NJ, USA). These enrichments remained with the mice throughout the experimental course, transferring through their weekly cage changes. Beginning on postnatal day 90, we randomly assigned males to either the Control or alcohol (EtOH) treatment groups and exposed them to either the Control treatment (water) or 10% ethanol (w/v; catalog# E7023, Millipore-Sigma, St. Louis, MO, USA). As described previously, [30] we employed a prolonged version of the Drinking in the Dark model, [50] which allows mice limited, voluntary access to the Control and EtOH treatments. Here, males were allowed access to the Control or EtOH treatments for a four-hour period that began four hours after the initiation of the dark cycle. We ensured identical treatment of Control males by switching between two identical water bottles. We eliminated males that dropped below 1.14g/kg (two standard deviations below the average fluid consumption of 1.7g/kg) for two consecutive weeks [30]. We continued the exposures for a six-week preconception phase and then maintained the exposures during the subsequent twelve-week breeding phase.

On postnatal day 83, we individually caged female mice and allowed them to acclimate to individual housing conditions for one week. We then randomly assigned postnatal day 90 females to the experimental (EtOH) (10% ethanol w/v) or Control treatment groups. Four hours after the initiation of the dark cycle, we replaced the water bottle of the female's home cage with the appropriate treatment bottle. We allowed female mice voluntary access to the Control or EtOH treatment for four hours and then returned their original water bottle to their home cage. We simultaneously exchanged the water bottles of Control and EtOH-exposed dams to ensure identical conditions.

At the end of each week, we recorded the weight of each mouse (g) and the amount of fluid they had consumed (g) and then calculated the weekly fluid consumption as grams of fluid consumed per gram of body weight. To remain consistent with clinical studies, [51] we converted this number to grams per kilogram (g/kg).

We initiated maternal exposures ten days before breeding dams to treated males. After seven to ten days of exposure, we synchronized female reproductive cycles using the Whitten method [52]. Then, after the daily Control or EtOH treatments, we placed a single female into the home cage of a treated male. After six hours, we confirmed matings by the presence of a vaginal plug and returned the female mice to their home cage. We ensured males rested for at least 72 hours before a subsequent mating. We subjected dams to minimal handling and maintained the EtOH and Control treatments until gestational day 10.5. We used a body weight gain of approximately 1.8g to diagnose pregnancy. As most women self-report the cessation of alcohol consumption after becoming pregnant, [53] we ceased the Control and EtOH treatments upon diagnosing pregnancy on gestational day 10.5. Thereafter, we provided dams with three nestlets, one Manzanita wood gnawing stick (catalog# W0016, Bio-Serv, Flemington, NJ, USA), and one gummy bone (catalog# K3585, Bio-Serv, Flemington, NJ, USA) and left pregnant females undisturbed with no handling or cage changes and allowed them to deliver their offspring. We resumed cage changes on postnatal day seven, maintaining the cage enrichments (one igloo, three extra nestles, one Manzanita wood gnawing stick, and one gummy bone) through the cage changes.

### Diethylnitrosamine (DEN) treatments

On the morning of postnatal day 14, we weighed male offspring, and IP injected a single dose of DEN (catalog# 73861; Millipore Sigma, Burlington, MA, USA) at a dose of 25mg/kg, adjusted to a total injection volume of 50ul, into each mouse. We treated control animals with an equivalent injection of saline alone. After the injection, we visually monitored mice for 24 hours and once every day until postnatal day 21, when we weaned and co-housed littermates. Thereafter, we continued to provide enrichments to the animal cages, including shelter tubes for males and igloos for the females, weighed offspring once a week during their regular cage changes, and provided *ad libitum* access to food and water.

### Offspring Dissection and tissue collection

At 24 weeks of age, we used isoflurane to induce anesthesia and performed retro-orbital blood collections. We then sacrificed the offspring using cervical dislocation and conducted a whole-body dissection, collecting the liver, spleen, pancreas, kidneys, adrenal glands, heart, thymus, and testes. During dissections, we weighed collected organs and either fixed tissue samples in 10% neutral buffered formalin (catalog# 16004-128, VWR, Radnor, PA, USA) or snap-froze the tissues on dry ice and stored them at -80C.

### Histology and immunohistochemistry

#### Frozen sectioning and Oil Red Staining

As described previously, we conducted frozen sectioning and oil red o staining [36]. Briefly, we dissected a central portion of the frozen liver and embedded it in Optimal Cutting Temperature (OCT) compound (catalog # 4583, Sakura Finetek, Torrance, CA, United States). We sectioned the OCT-embedded livers using a CM3050 S microtome-cryostat (Leica Biosystems, Deer Park, IL, United States) at 14µm. Then, we stained the liver sections using an Oil-Red-O kit (catalog# ab150678, Abcam, Boston, MA, United States) per the manufacturer's instructions. We randomly selected three areas of the same tissue section and imaged each area using a VS120 Virtual Slide Microscope (Olympus, Waltham, MA, United States).

#### Paraffin Sectioning for Sirius Red and H&E Staining

As described previously, [36] we washed fixed liver samples in PBS and processed them for histological analysis using a TP1020 Automatic Benchtop Tissue Processor (Leica Biosystems, Deer Park, IL, United States). After processing, we embedded the tissue in paraffin and sectioned it using an RM2255 Rotary Microtome (Leica Biosystems, Deer Park, IL, United States). Per the manufacturer's instructions, we stained liver sections using the Sirius Red (catalog# ab150681, Abcam, Boston, MA, United States) and Hematoxylin and Eosin (H&E) kits (catalog# ab2458800, Abcam, Boston, MA, United States). We randomly selected three areas of the same tissue section and imaged each area using the VS120 Virtual Slide Microscope (Olympus, Waltham, MA, United States).

#### Histological assessment

We sent blinded H&E slides to a board-certified veterinary pathologist (Jones-Hall) for histological assessment. We scored DEN-induced lesions between treatments using a modified NASH score system based on criteria described previously [54, 55]. Specifically, we scored macro- and microvesicular vacuolation (steatosis), hepatitis, and fibrosis (periportal, portal, and/or bridging), and for the presence/severity of nodular hyperplasia, scoring each lesion 0-3 (none, mild, moderate, severe) or 0-1 (for the presence (1) or absence (0) of adenoma(s) or hepatocellular carcinoma(s)).

#### Immunohistochemistry

We deparaffinized liver tissue sections in xylene and rehydrated the sections through decreasing grades of ethanol (100%, 95%, and 70%). We performed F4/80 and PCNA immunohistochemistry using the Alexa Flour™ 594 Tyramide Superboost™ Kit (catalog# B40922, ThermoFisher Scientific, Waltham, MA, USA). Briefly, we performed antigen retrieval using a heat retrieval process in citrate buffer (10mM sodium citrate buffer (tri-sodium citrate in water) with 0.05% Tween 20) overnight at 65°C. We blocked endogenous signals using a solution of 3% Hydrogen Peroxide and then incubated slides with protein block (catalog# ab64226, Abcam, Boston, MA, USA) at room temperature for one hour. We then incubated slides with rabbit anti-F4/80 or anti-PCNA antibodies (catalog# ab300421 and ab29, Abcam, Boston, MA, USA) overnight at 4°C. Subsequently, we washed slides in phosphate-buffered saline with Tween R20 solution and then incubated them with poly-HRP-conjugated secondary antibody at room temperature for one hour, followed by a tyramide amplification (catalog# B40922, ThermoFisher Scientific, Waltham, MA, USA) and staining with DAPI solution. We dried the slides and mounted them with Prolong Gold (catalog# P36934, ThermoFisher Scientific, Waltham, MA, USA).

### RNA isolation and reverse transcriptase quantitative Polymerase Chain Reaction (RT-qPCR)

As described previously, [36] we isolated RNA from the offspring's liver using the RNeasy Plus Mini Kit (catalog# 74136; Qiagen, Germantown, MD, USA) according to the manufacturer's instructions. We assessed RNA purity and concentration using a NanoDrop 2000 Spectrophotometer (Thermo Scientific, Waltham, MA, USA). We seeded approximately 1ug of RNA into a reverse transcription reaction using the High-Capacity cDNA Reverse Transcription Kit (catalog# 4368814; Thermo-Fisher, Waltham, MA, USA). Next, we determined the relative levels of candidate gene transcripts using the AzuraView GreenFast qPCR Blue Mix LR kit (catalog# AZ-2320: Azura Genomics, Raynham, MA, USA). We performed all reactions on a Bio-Rad CFX384 qPCR machine. Primer sequences are in Table 1.

**Table 1:** PCR primer sequences.

Gene	Forward	Reverse
**β-actin**	CCACCATGTACCCAGGCATT	CGGACTCATCGTACTCCTGC
**Ywhaz**	TTGATCCCCAATGCTTCGC	CAGCAACCTCGGCCAAGTAA
**Tgf-β**	CCGCAACAACGCCATCTATG	TTCCGTCTCCTTGGTTCAGC
**Timp1**	GCATGGACATTTATTCTCCACTGT	TCTCTAGGAGCCCCGATCTG
**Smad7**	CGGAAGTCAAGAGGCTGTGT	GACAGCCTGCAGTTGGTTTG
**Stat3**	GGGGTCACTTTCACTTGGGT	GACATCGGCAGGTCAATGGTA
**Nrf2**	CGAGATATACGCAGGAGAGGTAAGA	GCTCGACAATGTTCTCCAGCTT
**Gpx**	GAAGAACTTGGGCCATTTGG	TCTGCCTGGCTCCTGTTT
**Prx1**	GATCCCAAGCGCACCATT	TAATAAAAAGGCCCCTGAAAGAGAT
**Sod1**	GTGATTGGGATTGCGCAGTA	TGGTTTGAGGGTAGCAGATGAGT
**mtD-Loop**	TCCTCCGTGAAACCAACAA	AGCGAGAAGAGGGGCATT
**Tert**	CTAGCTCATGTGTCAAGACCCTCTT	GCCAGCACGTTTCTCTCGTT

### RNA isolation, sequencing, and informatic analysis

We isolated RNA from the liver using the RNeasy Plus Mini Kit (catalog# 74136; Qiagen, Germantown, MD, USA) according to the manufacturer's instructions. The isolated RNA samples were sent to Quick Biology (Pasadena, CA, USA), where we employed a total RNA library prep using rRNA depletion, followed by Illumina sequencing with 30M pair-reads. We trimmed the resulting raw fastq files with Trimmomatic [56], then employed FastQC [57], to perform quality control analysis and confirm the removal of adapter sequences. Next, using default parameters, we used RNA STAR [58] to map the reads to the Mus musculus reference genome (UCSC version GRCm39/mm39). We used the resulting mapped feature files in bam format and featurecounts [59] to generate a genes count table. We imported this table into R (R version 4.1.1 (2021-08-10)) for further analysis. We used DESeq2 [60] to generate the PCA plots and the list of differentially expressed genes (DEGs). We exported differentially expressed genes into the Ingenuity Pathway Analysis [61] software package and conducted pathway enrichment analysis. We generated volcano plots in GraphPad using a q-value<0.05 and a log-fold change of 1.5. We deposited transcriptomic data under the GEO Accession GSE273675.

### Protein Extraction and Western Immunoblot Analysis

We extracted proteins from frozen liver samples using RIPA buffer and quantified protein levels using a BCA Protein Assay Kit (catalog# 23227 Thermo Scientific, Waltham, MA, USA). We loaded 20µg of protein on an 8% SDS polyacrylamide gel and transferred proteins onto a PVDF membrane for 2 hours at 60V. We blocked membranes in 5% milk for one hour and then blotted membranes using antibodies recognizing CYP2E1 (catalog# ab28146, Abcam, Boston, MA, United States) at 4°C overnight. We washed blotted membranes in TBST for one hour and then added Anti-Rabbit (Catalog #926-80011, Li-Cor, Lincoln, NE, USA) secondary antibody. We imaged the blots using the Li-cor chemiluminescence system (catalog #926-95000, Li-Cor, Lincoln, NE, USA) with the BioRad Chemidoc MP. We performed a densitometric analysis of bands using ImageJ.

### Assays and ELISAs

We determined plasma Alanine Transaminase (ALT) and Aspartate Aminotransferase (AST) activity using an Alanine Transaminase Activity Assay kit (catalog# ab105134, Abcam, Boston, MA, United States) and Aspartate Aminotransferase Activity Assay kit (catalog# ab105135, Abcam, Boston, MA, United States) per the manufacturer's instructions. We quantified the levels of IL-6 and TNF-α in the livers of male mice using an IL-6 ELISA (catalog# ab100713, Abcam, Boston, MA, United States) and TNF-α ELISA (catalog# ab108910, Abcam, Boston, MA, United States) per the manufacturer’s instructions. We determined the NAD+/NADH ratio using the NAD/NADH Assay Kit (catalog# ab65348, Abcam, Boston, MA, USA) following the provided protocol. We measured mitochondrial complex 1 (catalog# ab109721, Abcam, Boston, MA, USA) and Malate Dehydrogenase (catalog# ab119693-1001, Abcam, Boston, MA, USA) enzymatic activity using commercially available colorimetric activity assays following the manufacturer's protocols. We determined the levels of reactive oxygen species (ROS) in the liver using a Lipid Peroxidation (MDA) Assay (catalog# ab118970, Abcam, Boston, MA, United States) and 8-hydroxy 2-deoxyguanosine (8-OHdG) ELISA (catalog# ab201734, Abcam, Boston, MA, United States) per manufacturer’s instructions.

### Data handling and statistical analysis

We subjected all data generated during this study to the data management practices and statistical analyses described previously [36, 42]. Briefly, we recorded our initial observations by hand and then inserted these measurements into Google Sheets or Microsoft Excel. We calculated the total daily caloric intake during gestation by multiplying the grams of food consumed between gestational Days 0 and 10 by the energy density of the supplied diet. We then determined the calories derived from EtOH by multiplying the grams of fluid consumed by 0.1 (10% EtOH) and 7 Kcal/g ethanol. To analyze offspring organ weights, we normalized the values for organ weights to the animal's total body weight at sacrifice. During dissections, we recorded the number of visible tumors on each liver. We determined tumor incidence by assigning a value of “1” to animals with visible tumors and “0" to those without, then input these data into a Chi-square table for analysis. To determine tumor multiplicity, we counted the total number of visible tumors and input these values into a separate Chi-square table. Animals with no visible tumors received a score of 0 in both comparisons.

For the analysis of Oil-Red-O and Sirius Red histological stains, we used Photoshop to standardize the levels, parameters, and saturation of each image. We then converted the color scheme to black and white, with the red color of lipid droplets adjusted to black and the surrounding tissue showing gray. We then imported the images into ImageJ, used the threshold property to cover the black stain, and excluded the gray area. We then analyzed images for staining intensity and expressed the quantification as a percentage of the total tissue area positively stained for lipids. We averaged three percentages for each sample to obtain the final % of tissue area. To quantify F4/80-positive cells, we imaged liver sections at 20X magnification using an Olympus BX61 microscope with a Hamamatsu ORCA-ER camera (Hamamatsu Photonics, Shizuoka, Japan). Using the Slidebook software (Intelligent Imaging Innovations (3i), Denver, CO, USA), we manually determined the area of a region of interest (mm2) and normalized the number of F4/80-positive cells to the total area. We randomly selected three areas of the same tissue section and averaged the positive area ratio for further analysis. To quantify total hepatic PCNA staining intensity, we imaged liver sections at 20X magnification using an Olympus BX61 microscope with a Hamamatsu ORCA-ER camera (Hamamatsu Photonics, Shizuoka, Japan), then imported the images into ImageJ and normalized total PCNA area by total nuclear area.

We normalized gene expression values by importing the RT-qPCR replicate cycle threshold (Ct) values for each gene of interest into Microsoft Excel and normalized them to the geometric mean of reference genes β-actin and Ywhaz. We then used the -ΔΔCT method [62] to calculate the relative fold change for each biological replicate. We conducted histological analysis using sample blinding, but the molecular analysis of tissue samples was not.

We have moved away from categorizing our results as significant or not significant based solely on a p-value and now use statistical testing as a means of grading the strength of evidence against the null hypothesis [63]. We consider p-values above 0.01 to be strong evidence for an effect, while p-values between 0.1 and 0.01 provide moderate evidence of an effect [64]. Here, we report the exact p-values for moderate evidence and, to avoid cluttering graphs, use ** (P < 0.01), *** (P < 0.001), and **** (P < 0.0001) to denote strong evidence of an effect.

**Figure 1.**
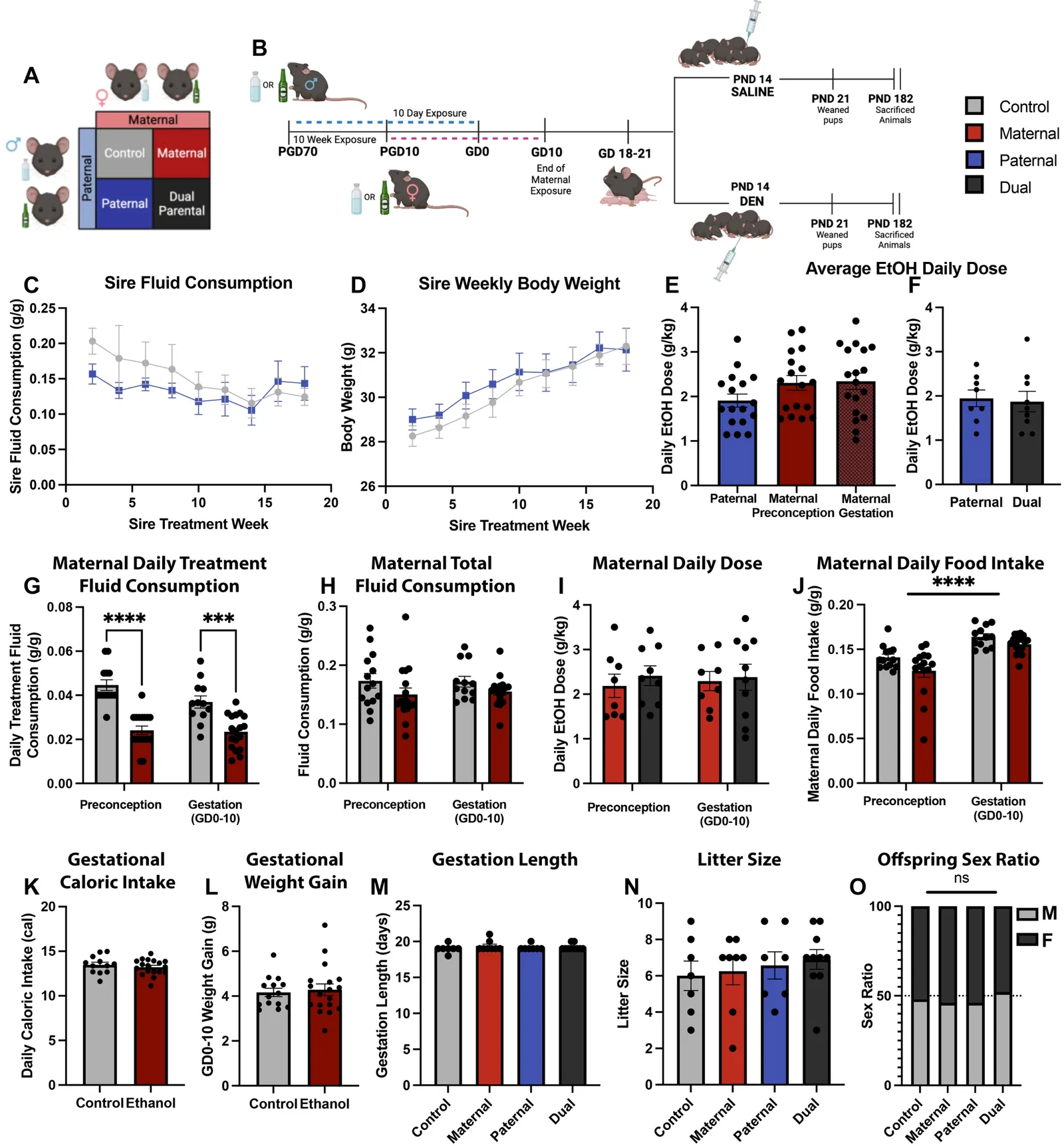
**A multiplex mouse model to determine the impacts of maternal and paternal periconceptional alcohol use on offspring predisposition to toxicant-induced hepatocellular carcinoma**. (**A**) Visual representation of the 2x2 factorial experimental paradigm. (**B**) Schematic representation of the experimental design we employed to study the impacts of parental alcohol consumption on offspring predisposition to toxicant-induced hepatocellular carcinoma. (**C**) Weekly fluid consumption and (D) weight gain of Control (n=9) and EtOH-exposed (n=9) sires across the experimental course, including the 6-week preconception and 12-week breeding phases. (**E**) Average daily dose of EtOH compared between males (n=17) and females (n=18) and between the preconception and gestation phases. We calculated the average daily dose by multiplying the average weekly fluid consumption (g/g) by 0.10 (10% EtOH), dividing this number by 7 (days), and converting it to g/kg. (**F**) Average daily dose of EtOH compared between sires in the PatExp (n=8) and DualExp (n=9) treatment groups. Comparison of maternal (G) average daily fluid intake during the four-hour exposure window and (H) total daily fluid intake between the Control and EtOH treatments during the preconception and gestational treatment phases (n=12-14). (**I**) Comparison of average daily EtOH dose between females within the MatExp (n=8) and DualExp (n=10) treatments. (**J**) Comparison of maternal average daily food consumption between Control (n=14) and EtOH (n=18) treatments during the preconception and gestational treatment phases. (**K**) Comparison of maternal daily gestational caloric intake, including calories derived from EtOH, between the Control (n=12) and EtOH (n=18) treatments. (**L**) Comparison of dam weight gain between gestational days 0 and 10 of pregnancy. Comparison of (M) gestation length, (N) litter size (n=7-10), and (O) the ratio of male and female offspring between the treatment groups. We compared treatments using a one- or two-way ANOVA or Chi-square analysis. Data represent mean ± SEM; we include p-values between 0.1 and 0.01 above each figure, while we have used ** to denote P < 0.01, *** P < 0.001, and **** P < 0.0001.

For statistical analysis, we transferred the collected datasets into GraphPad Prism 10 (RRID:SCR_002798, GraphPad Software Inc., La Jolla, CA, USA). We first employed the ROUT test (Q = 1%) to identify outliers and verified equal variance using either the Brown-Forsythe or F testing. If data passed normality and variance testing (alpha = 0.05), we employed either an unpaired, parametric (two-tailed) t-test or a One-way or Two-way ANOVA. We then used Tukey's post hoc test to compare each treatment or Dunnett’s test to compare all treatments to the Control. For comparisons of mitochondrial enzymatic activity, we used repeated measures ANOVA contrasting treatment effects across time. If, however, the collected datasets failed the test for normality or we observed unequal variance, we ran a Kruskal-Wallis test followed by Dunn's multiple comparisons test. We present detailed descriptions of each statistical test and the sample sizes for each Figure in Supplementary Table 1.

## RESULTS

### A multiplex mouse model to determine the impacts of parental alcohol use on offspring predisposition to toxicant-induced hepatocellular carcinoma.

Because mouse handling and gavage potently induce the stress response, [65] which directly affects epigenetic programming in oocytes and sperm, [66] we employed the voluntary *Drinking in the Dark* method, [50] allowing animals to consume alcohol according to their preference, and subjected mice to minimal handling. To test the hypothesis that parental alcohol exposure increases offspring susceptibility to environmentally-induced hepatocellular carcinoma, we employed our established multiplex mouse model [36, 42] that utilizes a 2x2 mating paradigm to compare offspring obtained from Control, maternal (MatExp), paternal (PatExp), and dual-parental (DualExp) models of alcohol exposure (Fig. 1A).

We exposed postnatal day 90 C57BL6/J males to water (Control) or alcohol (EtOH) for a six-week preconception phase, then maintained exposures during the subsequent twelve-week breeding phase (Fig. 1B). We did not observe any differences in weekly fluid consumption or weight gain between males in the Control and EtOH treatment groups during either the preconception or breeding phases (Fig. 1C-D). EtOH-exposed sires received an average daily dose of 1.91g/kg (Fig. 1E), which, in our previous studies, [30] correlated with plasma alcohol concentrations of 100-150mg/dL (1.25-1.8 x the legal limit in the US) and induced heritable, epigenetic changes in offspring fetoplacental growth. We did not observe any differences in daily dose between males in the PatExp and DualExp treatments (Fig. 1F). After six weeks of exposure (approximately one complete spermatogenic cycle), we bred Control and EtOH-exposed males to Control or EtOH-exposed females (Fig. 1A).

We exposed female mice to water (Control) or EtOH for an initial preconception period, approximately 7-14 days, but ceased experimental treatments after pregnancy diagnosis on gestational day ten (Fig. 1B). We designed this paradigm to mimic human behaviors, where most women self-report the cessation of alcohol consumption after a pregnancy diagnosis [53]. Although EtOH-exposed dams exhibited decreased fluid consumption during the four-hour treatment window, we did not observe any differences in total daily maternal fluid consumption between the Control and EtOH treatments (Fig. 1G-H). Dams received an average daily dose of 2.30g/kg during the preconception phase and 2.34g/kg during gestational days 0-10 (Fig. 1E). We did not observe any differences in maternal average daily EtOH dose between the preconception or pregnancy phases (Fig. 1E), nor did we identify any differences in average daily EtOH dose between dams in the MatExp or DualExp treatment groups during either the preconception or gestational phases (Fig. 1I).

In toxicological studies examining maternal exposures, pair-feeding is an additional treatment employed to account for the adverse effects of toxicants on maternal caloric intake during pregnancy [67, 68]. To explore the necessity of this additional treatment in our model, we examined the effects of alcohol on maternal daily food and total caloric intake during gestation. We did not observe any treatment effects on maternal daily food intake between the Control and EtOH treatments (Fig. 1J). We did, however, observe increased food consumption during pregnancy compared to the preconception window, as anticipated (Fig 1J). Notably, when we accounted for the additional calories derived from EtOH, we still did not observe any differences in maternal gestational daily caloric intake between treatments (Fig. 1K), nor did we observe any impacts of EtOH on maternal weight gain during pregnancy (Fig. 1L). As we did not observe any measurable impacts on maternal food consumption, daily caloric intake, or weight gain, we did not implement a pair-fed control.

After diagnosing pregnancy on gestational day ten, we ceased all treatments and animal handling and allowed dams to deliver their offspring. We did not observe any treatment effects on gestation length, litter size, or offspring sex ratio between the experimental treatments (Fig. 1M-O).

### Parental alcohol use increases tumor number and diameter in a mouse model of DEN-induced liver injury.

To determine if parental alcohol use predisposes offspring to toxicant-induced carcinogenesis, we used the well-established model of Diethylnitrosamine (DEN)-induced carcinogenic liver injury [69]. As DEN treatments predominantly impact males [43] and chronic liver disease is more common in men than women, [70] we focused our analysis on male offspring. We randomly assigned male offspring to either the Saline (control) or DEN treatments (Fig. 1B). Using an IP dose of 25mg/kg DEN or saline alone, we treated the male offspring on postnatal day 14 and monitored their subsequent growth and development.

**Figure 2.**
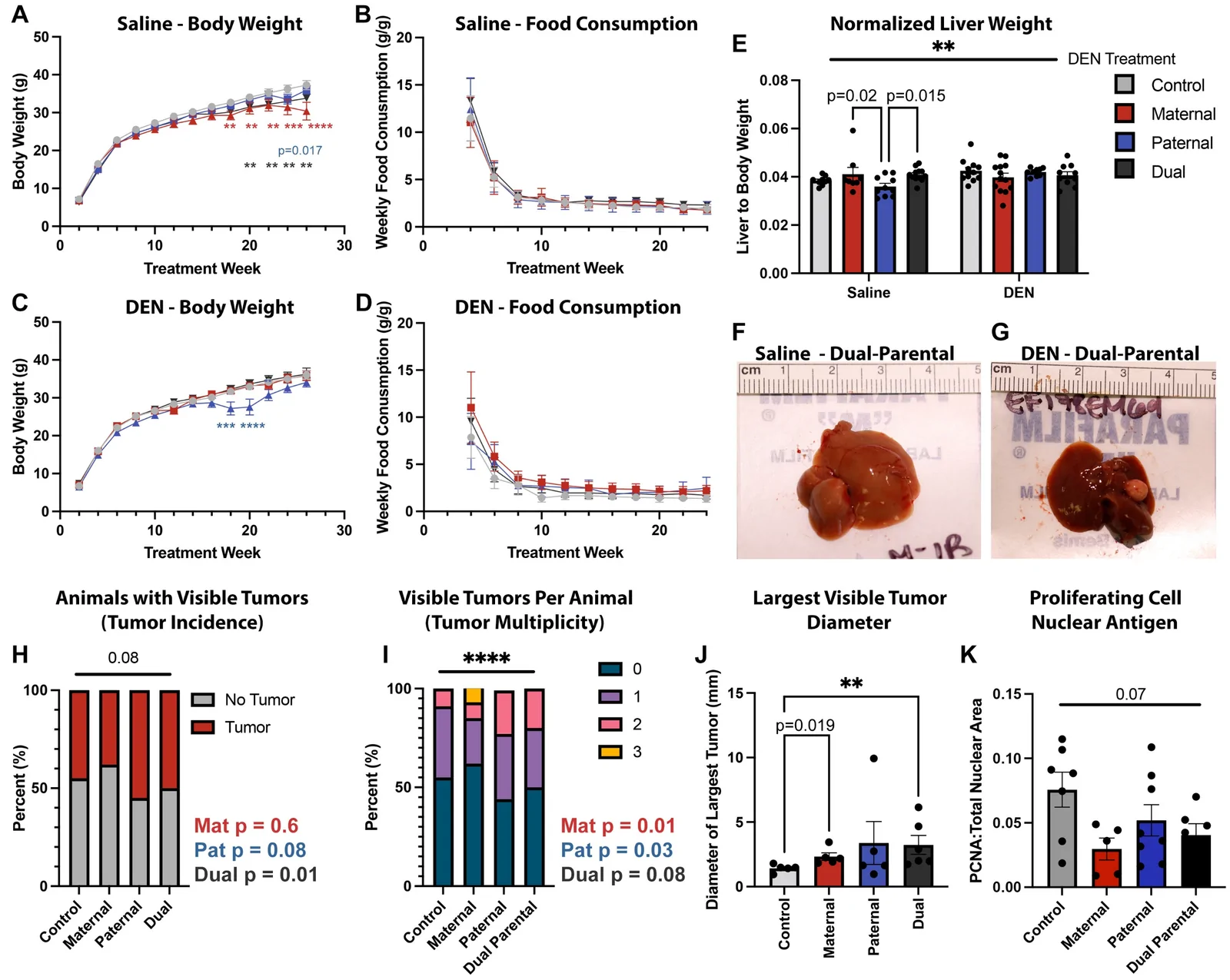
**Parental alcohol use increases tumor incidence, number, and diameter in a mouse model of DEN-induced liver injury**. We used a repeated measures ANOVA to compare the impacts of parental alcohol treatment on male offspring (A) body weight and (B) food consumption in the Saline treatment group (n=9-13). Comparison of the impacts parental alcohol exposure has on DEN-treated male offspring (C) body weight and (D) food consumption over 26 weeks of life (n=9-13). (**E**) Comparison of the impacts of parental alcohol exposure on bodyweight-normalized liver weights between the Saline and DEN treatment groups (n=9-13). Representative images of livers taken from (F) Saline and (G) DEN-treated Dual-Parental male offspring at the time of dissection. We compared the (H) number of mice with visible tumors, (I) number of visible tumors per mouse (n=9-13), and (J) diameter of the largest visible tumor between the parental alcohol treatment groups in DEN-treated offspring (n=5-6). (**K**) We used immunohistochemistry to compare hepatic Proliferating Cell Nuclear Antigen (PCNA) staining intensity in the DEN treatment group (n=7,5,8,6). We analyzed datasets using repeated measures ANOVA or a two-way ANOVA followed by Tukey's post-hoc test or Chi-squared analysis. Data represent mean ± SEM; we include p-values between 0.1 and 0.01 above each figure, while we have used ** to denote P < 0.01, *** P < 0.001, and **** P < 0.0001.

Despite no measurable changes in offspring food consumption, we observed evidence of modest declines in offspring body weights for the MatExp and DualExp preconception treatment groups within the saline treatment (Fig. 2A-B). In contrast, we only observed a transient decline in offspring body weights in the preconception PatExp treatment group in the DEN-treated offspring but, again, did not observe any impact on food consumption (Fig. 2C-D). Notably, none of the treatment-related declines in body weight exceeded the 20% threshold, requiring the implementation of an early humane endpoint [71].

Because we anticipated parental alcohol exposure would increase offspring susceptibility to DEN-induced tumorigenesis, we elected to sacrifice offspring at six months of age before progression into the end stages of liver disease. At sacrifice, we observed an effect of the DEN treatment on the liver-to-body weight ratio compared to Saline males across all treatment groups (Fig. 2E). During dissection, we noted that the livers of DEN-treated males were pale in comparison to Saline males, suggesting increased lipid accumulation. In the DEN treatment group, we observed modest to strong evidence of an increased incidence of cancer (*i.e*., the number of tumor-positive animals) in the offspring of the PatExp and DualExp treatments compared to Control offspring (Fig. 2F-H), suggesting a more robust response to the DEN carcinogen. Strikingly, in the DEN treatment group, offspring from each of the MatExp, PatExp, and DualExp treatments exhibited modest to strong evidence of increased tumor multiplicity (increased number of visible tumors) compared to the offspring of Controls (Fig. 2I). When comparing the diameter of the largest observable tumor in DEN-exposed offspring, we observed modest evidence of increased tumor diameter within MatExp offspring but strong evidence of increased tumor diameter in the DualExp treatment compared to Controls (Fig. 2J). We did not identify any observable tumors in the Saline treatment group. Although we identified modest evidence of an overall inhibitory effect of parental alcohol exposure on total hepatic Proliferating Cell Nuclear Antigen (PCNA) staining in the DEN treatment group, multiple testing did not identify any significant differences (Fig. 2K).

### Maternal and paternal alcohol use interact in driving the progression of toxicant-induced liver disease.

**Figure 3.**
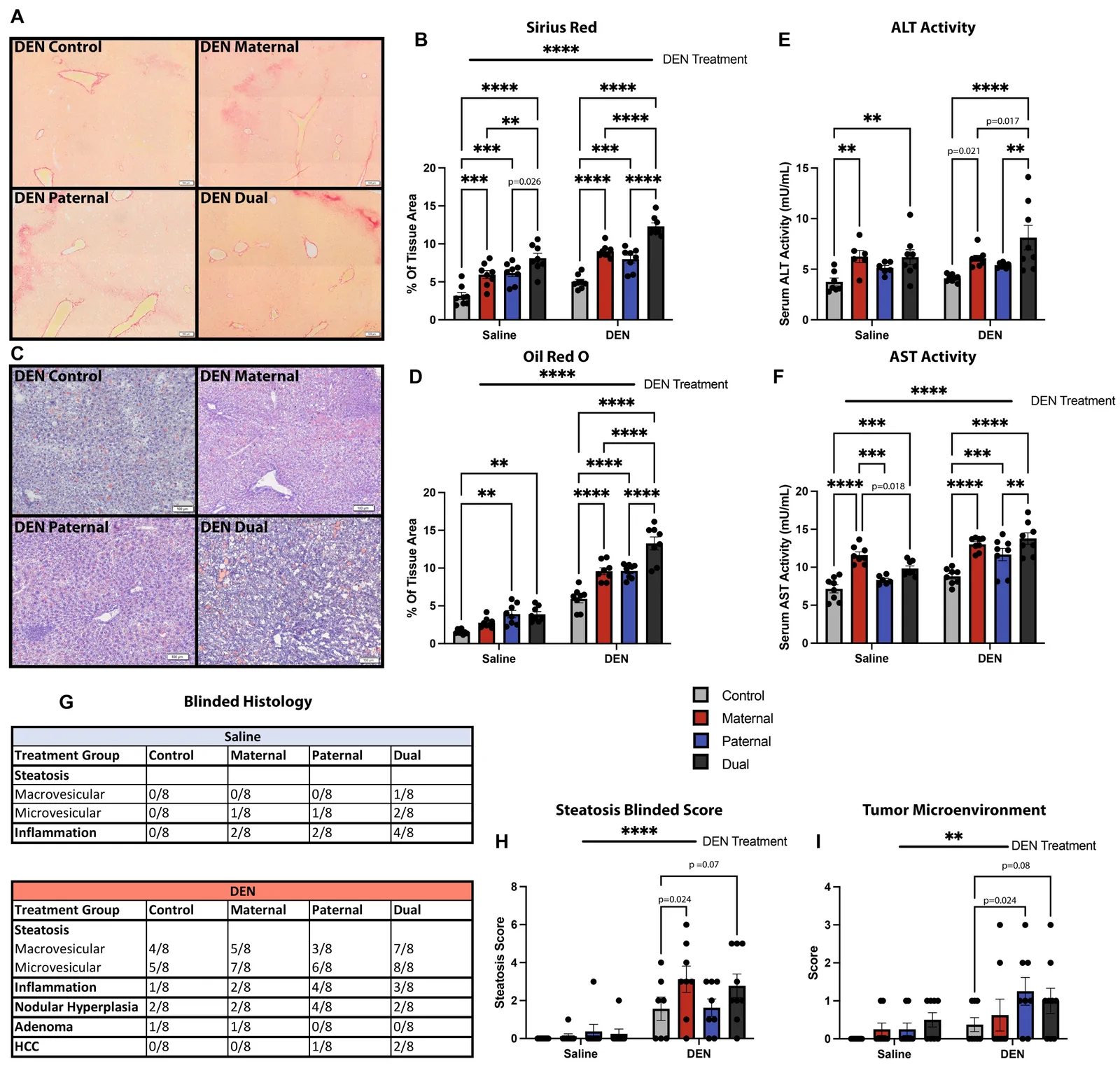
**Maternal and paternal alcohol use interact in driving the progression of toxicant-induced liver disease**. (**A**) Representative images and (B) quantification of male histological sections stained with Picrosirius Red (n=8). C) Representative images and (D) quantification of male histological sections stained with Oil Red-O (n=8). Comparison of (E) alanine transaminase (ALT) (n=8) and (F) aspartate transaminase (AST) (n=8) between treatments. (**G**) Tables comparing the histological scores between the preconception treatment groups (n=8). Quantification of histological scores assessing markers of (H) steatosis and (I) an inflammatory tumor microenvironment between treatment groups. We used a two-way ANOVA followed by Tukey's post-hoc test to compare treatment groups. Data represent mean ± SEM; we include p-values between 0.1 and 0.01 above each figure, while we have used ** to denote P < 0.01, *** P < 0.001, and **** P < 0.0001.

To understand the pathophysiological basis of the increased tumor incidence, number, and size, we first examined histological measures of liver health. Picro-Sirius red (Fig. 3A-B) and oil red O (Fig. 3C-D) staining revealed an adverse effect of the DEN treatment and a marked increase in histological indicators of fibrosis and steatosis, respectively, across all parental alcohol treatment groups. Next, we assessed clinical markers of liver damage between treatments by measuring circulating alanine transaminase (ALT) and aspartate transaminase (AST) activity. In the Saline treatment, we observed increased ALT and AST activity in MatExp and DualExp treatments (Fig. 3E-F). In the DEN treatment, we observed increased plasma ALT and AST activities across all parental alcohol treatment groups (Fig. 3E-F). Notably, for all histological measures and ALT and AST activities in the DEN treatment group, we observed an interaction between the MatExp and PatExp treatments, with outcomes in the DualExp offspring exceeding those induced by either maternal or paternal alcohol use alone.

We then stained tissue sections with H&E and sent slides to a board-certified pathologist for grading and staging of histological lesions, scoring slides for inflammation, steatosis, fibrotic lesions, and cancer progression, following the non-alcoholic steatohepatitis (NASH) score system [55] (Fig. 3G). This analysis identified increased steatosis, including increased macrovesicular and microvesicular steatosis in the MatExp and DualExp offspring in the DEN treatment (Fig. 3G-H). We then assessed histological measures indicative of tumor-promoting microenvironment, including assessment of inflammation, nodular hyperplasia, adenomas, and HCC. This analysis identified modest evidence of an increased tumor-promoting microenvironment in the PatExp and DualExp offspring in the DEN treatment (Fig. 3I). Collectively, these data indicate that offspring of alcohol-exposed parents exhibit increased liver steatosis, fibrosis, and inflammation, priming the hepatic environment to support toxicant-induced tumor formation.

### Parental alcohol exposure disrupts the transcriptional control of mitochondrial complex I and Transforming Growth Factor beta (TGF-β) signaling.

**Figure 4.**
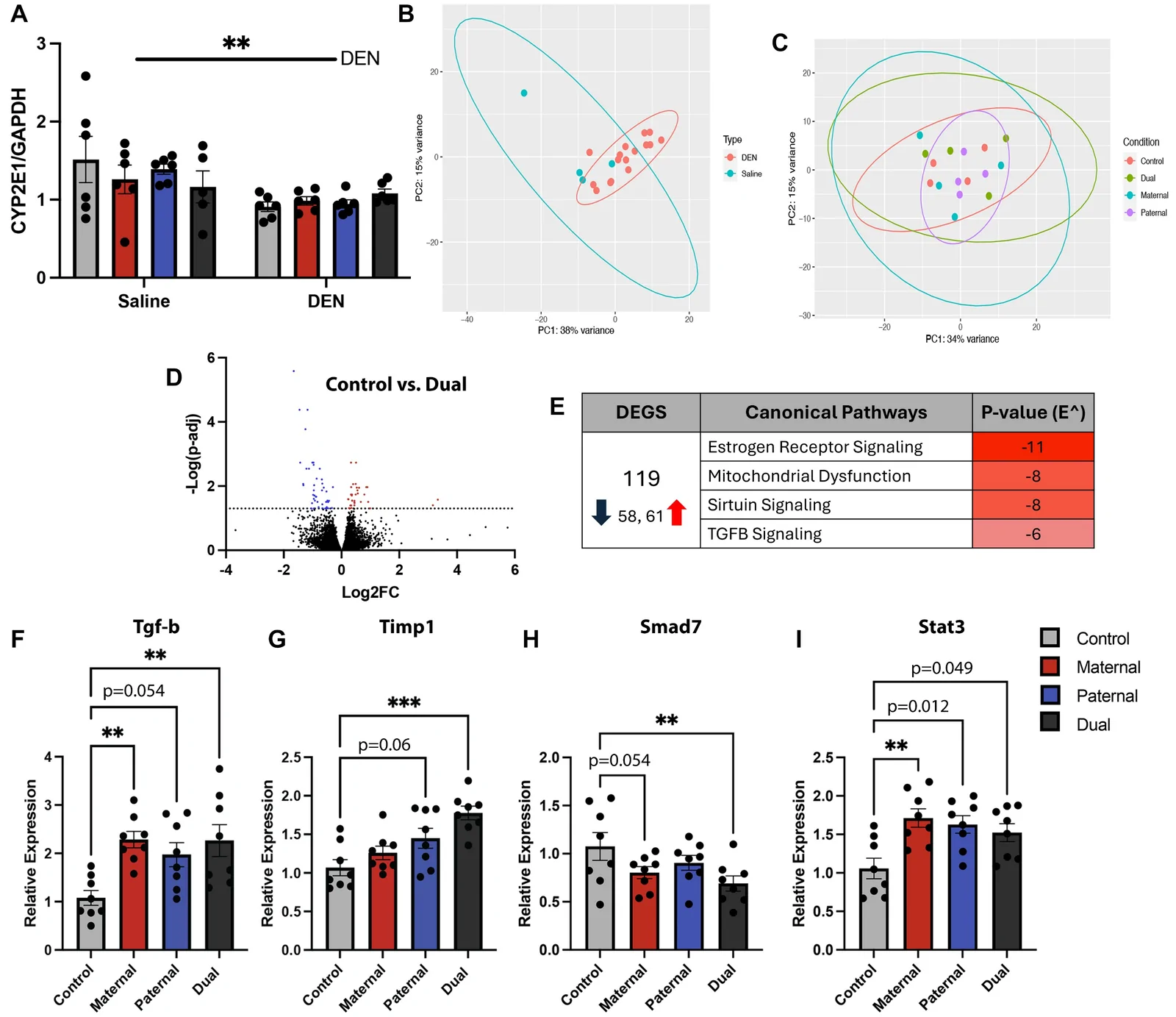
**Transcriptomic analysis reveals that parental alcohol exposures cause lasting disruptions in mitochondrial function and Transforming Growth Factor beta (TGF-β) signaling**. (**A**) Quantification of western blot analysis of cytochrome P450 enzyme CYP2E1 protein abundance between treatment groups (n=6). Principal component analysis comparing the transcriptomic profiles of livers isolated from (B) Saline- and DEN-treated offspring and (C) offspring of alcohol-treated parents within the DEN-treated cohort (n=4 per group). (**D**) Volcano plot contrasting down- and up-regulated genes (log- 1.5-fold change, q<0.05) in the DualExp treatment group. (**E**) Integrated pathway analysis comparing differently expressed genes between Control and DualExp offspring within the DEN-treated cohort (log 1.5-fold change, p<0.05). We used reverse transcriptase quantitative polymerase chain reaction (RT-qPCR) to compare transcripts encoding (F) the Tgf-β1 ligand, (G) Timp1, (H) Smad7, and (I) Stat3 between the parental treatment groups in the DEN cohort (n=8). We used a one-way ANOVA followed by Tukey's post-hoc test to compare treatments. Data represent mean ± SEM; we include p-values between 0.1 and 0.01 above each figure, while we have used ** to denote P < 0.01, *** P < 0.001, and **** P < 0.0001.

Previous studies examining toxicant-induced paternal effects on offspring have described increased expression of genetic factors promoting xenobiotic metabolism, including the cytochrome P450 enzyme CYP2E1 [72, 73]. Importantly, CYP2E1 is one of the core factors involved in alcohol detoxification, and increased CYP2E1 activity correlates with increased susceptibility to DEN-induced hepatocellular carcinoma [48, 74]. However, although we identified reduced CYP2E1 levels in DEN-exposed offspring compared to the Saline controls, western blotting did not identify any differences in CYP2E1 protein abundance between the parental treatment groups (Fig. 4A).

To better understand the molecular changes driving the observed histological indicators of liver disease, we isolated RNA and conducted deep sequencing of the hepatic transcriptome (n=4). Principal component analysis separated Control Saline samples from all other treatments within the DEN cohort (Fig. 4B). When we compared samples filtering for a log-fold change of 1.5 and q-value of 0.05, we identified 35 differentially expressed genes between Saline-Control and DEN-Control samples. When we relaxed these criteria and sorted genes using only a q-value of 0.1, we identified 563 differentially expressed genes. Aligning with previous studies describing deficits in mitochondrial respiration in DEN-induced tumors [44-47], comparisons of Saline-Control to DEN-Control and Saline Control to DEN-Paternal offspring identified differential expression of numerous members of mitochondrial complex I (Supplementary Fig. 1). Although these candidate genes exhibited small magnitude changes in expression, these same genes also appeared in our previous studies examining placentae derived from the offspring of alcohol-exposed males [29, 35, 30-32] and include both mitochondrial and nuclear transcripts encoding mitochondrial complex I, such as mt-Nd4, mt-Nd5, and Ndufa7. Unexpectedly, we did not identify as many members of mitochondrial complex I when comparing transcriptomes derived from Saline-Control to DEN-Maternal or DEN-Dual offspring.

In contrast to the comparisons between the Saline and DEN treatments, principal component analysis failed to identify a clear separation between the alcohol treatments within the DEN exposure group (Fig. 4C), suggesting that the parental exposures exert very modest impacts on the transcriptional control of gene expression in the adult stage. When making comparisons within the DEN treatment, comparing treatments to the DEN-Control, we identified a small number of differentially expressed mitochondrial and nuclear genes (log 1.5-fold change, q<0.05), with 17 differentially expressed genes in comparisons of MatExp offspring to Controls, 22 when comparing PatExp offspring to Controls, and 80 between DualExp offspring and Controls (Fig. 4D). We identified one overlapping gene between the MatExp, PatExp, and DualExp treatments, CCAAT enhancer binding protein beta (*Cebpb*), encoding a basic leucine zipper domain transcription factor regulating genes involved in immune and inflammatory responses. When we relaxed the stringency of our transcriptomic analysis to no longer account for false discovery (log 1.5-fold change, p<0.05) and used these expanded gene lists to conduct gene enrichment analysis, we identified alterations in pathways regulating oxidative phosphorylation, mitochondrial function, and Sirtuin signaling (Fig. 4E), similar to gestational day 16.5 offspring placentae [29, 35, 30-32]. Notably, we also identified modest to strong evidence of altered transforming growth factor beta (TGF-β) signaling, including an increase in the Tgf-β1 ligand, tissue inhibitor of metalloproteinases 1 (Timp1), Signal transducer and activator of transcription 3 (Stat3), and decreased expression of the Tgf-β negative regulator Mothers against decapentaplegic 7 (Smad7) (Fig. 4F-I). In sum, these observations suggest that both parental alcohol use and early-life DEN exposure cause modest changes in transcripts encoding mitochondrial complex I and elevations in TGF-β signaling.

### Parental alcohol exposures disrupt offspring mitochondrial complex I activity, inducing a persistent signature of oxidative stress.

**Figure 5.**
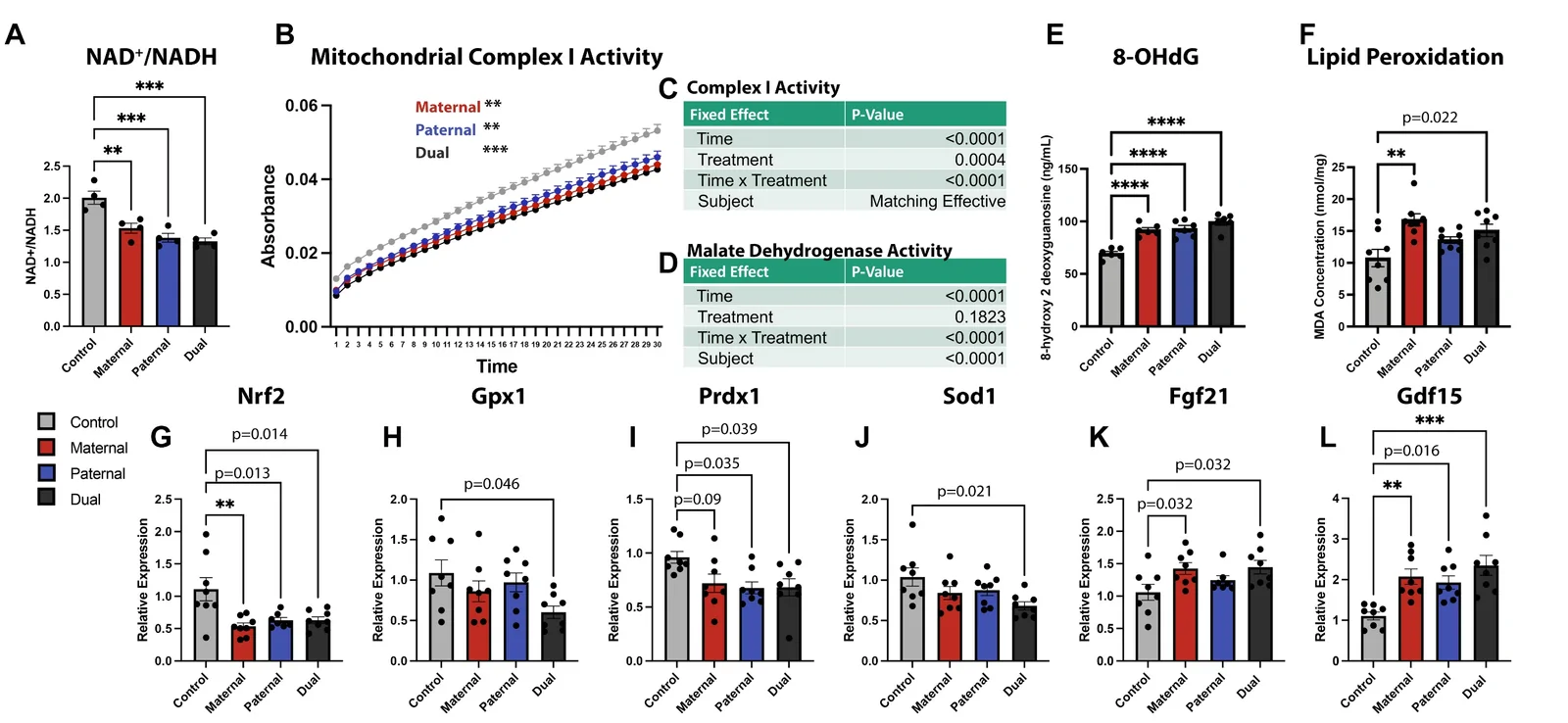
**Parental alcohol exposures heritably disrupt mitochondrial complex I activity in the DEN-exposed mice, causing persistent mitochondrial stress and transcriptional suppression of the antioxidant response in the offspring liver**. (**A**) Comparison of the NAD+/NADH ratio between the parental treatment groups in DEN-exposed offspring (n=4). We used a colorimetric assay to measure (B) mitochondrial OXPHOS complex I enzymatic activity over time and a repeated measures ANOVA to compare (C) Complex I and (D) Malate Dehydrogenase activities between treatments (n=8). We used ELISA assays to indirectly determine the levels of ROS by measuring (E) 8-OHdG and (F) MDA between treatment groups (n=6). We used RT-qPCR analysis to compare transcripts encoding genes involved in the antioxidant response between treatment groups, including (G) Nuclear Factor Erythroid 2-Related Factor 2 (Nrf2), (H) Glutathione Peroxidase 1 (Gpx1), (I) Peroxiredoxin 1 (Prdx1), (J) Superoxide Dismutase 1 (Sod1), (K) Fibroblast growth factor 21 (FGF21), and (L) Growth and differentiation factor 15 (Gdf15) (n=8). We used a one-way ANOVA followed by Tukey's post-hoc test to compare treatments. Data represent mean ± SEM; we include p-values between 0.1 and 0.01 above each figure, while we have used ** to denote P < 0.01, *** P < 0.001, and **** P < 0.0001.

Our previous studies examining the offspring of alcohol-exposed males have reported compromised transcriptional control of mitochondrial function and oxidative phosphorylation that first appears during fetal development and persists into middle age [29, 35, 30-32, 36]. Furthermore, in middle-aged adult offspring, we observed decreased conversion of cellular NADH to NAD+ and a reduced NAD+/NADH ratio across the MatExp, PatExp, and DualExp treatments. NADH oxidation to NAD+ occurs in mitochondria by the action of mitochondrial complex I [75]. Consistent with our studies examining aged offspring (42 weeks), we find that 26-week-old MatExp, PatExp, and DualExp offspring in the DEN treatment group exhibit strong evidence of a decreased NAD+/NADH ratio (Fig. 5A). Significantly, this reduced NAD+/NADH ratio correlates with robust evidence of decreased mitochondrial complex I activity across all treatments (Fig. 5B-C). In contrast, we do not identify any impacts of parental alcohol exposure on Malate Dehydrogenase 2 (MDH2) activity (Fig. 5D), indicating that the decline in the NAD+/NADH ratio is most likely linked to compromised mitochondrial complex I activity and not disruption of the citric acid cycle (MDH2 activity).

Compromised mitochondrial complex I activity and a decreased NAD+/NADH ratio are associated with an increased production of reactive oxygen species [75]. Consistently, we identified strong evidence of increased levels of DNA/RNA oxidative damage (8-hydro-xydeoxyguanosine (8-OHdG)) (Fig. 5E) and modest to strong evidence of increased lipid peroxidation (Fig. 5F) (malondialdehyde concentration), both reliable indicators of hepatic oxidative stress [76].

Cellular redox imbalance activates TGF-β signaling pathways, promoting inflammation, disrupting immune signaling, and driving cancer pathogenesis [77, 78]. Specifically, increased ROS production potently activates TGF-β1 signaling (Fig. 4E), driving tissue fibrosis and the suppression of antioxidant enzymes. Transcriptional suppression of antioxidant enzymes, in turn, generates more reactive oxygen species, further activating TGF-β1 signaling, forming a vicious positive feed-forward cycle [77]. Using RT-qPCR, we observed modest evidence of reduced transcripts encoding multiple antioxidant enzymes, including Nuclear Factor Erythroid 2-Related Factor 2 (Nrf2), Glutathione Peroxidase 1 (Gpx1), Peroxiredoxin 1 (Prdx1), and Superoxide Dismutase 1 (Sod1) in the DEN treatment group, predominantly in DualExp offspring (Fig. 5G-J). Persistent oxidative stress reinforced by the suppression of the antioxidant response causes the accumulation of unfolded proteins in the mitochondrial matrix, activating the integrated stress response. In response, the cell upregulates Fibroblast growth factor 21 (Fgf21) and Growth and differentiation factor 15 (Gdf15) [79]. Notably, the levels of these mitokines positively correlate with liver cancer development, progression, and metastasis [79]. Using RT-qPCR, we identified modest evidence of increased transcription of Fgf21 and strong evidence of transcriptional up-regulation of Gdf15 (Fig. 5K-L).

### Parental alcohol exposures program immune dysfunction and support the development of an inflammatory precancerous microenvironment.

**Figure 6.**
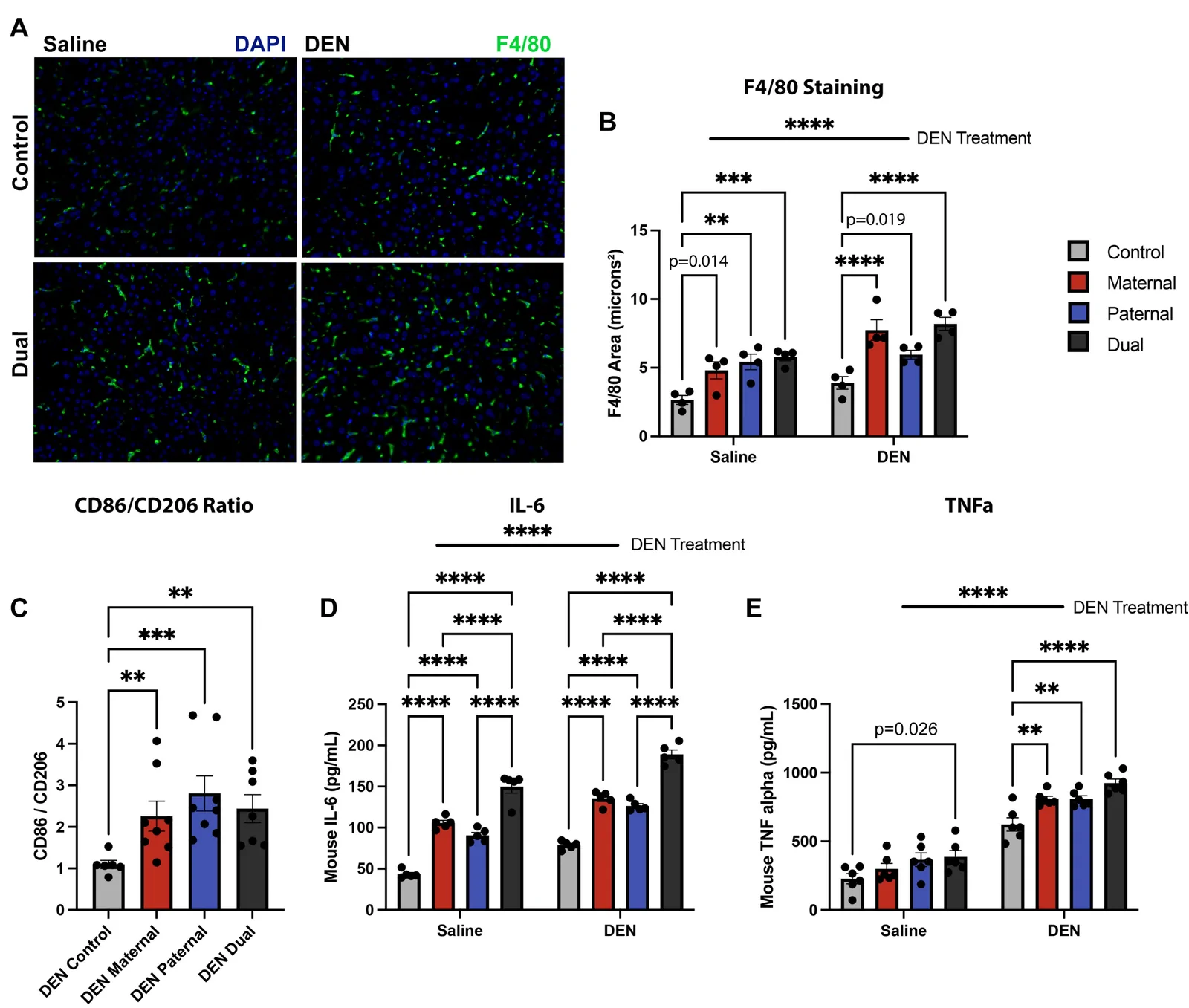
**Parental alcohol exposures program immune dysfunction, supporting the development of an inflammatory precancerous microenvironment**. (**A**) Representative images of male liver immunohistochemistry staining using an antibody recognizing the macrophage marker F4/80. (**B**) Quantification of F4/80 staining compared between treatment groups (n=4). (**C**) We used RT-qPCR to compare the ratio of CD86 to CD206 and determine the prevalence of proinflammatory macrophages (n=8). We used ELISA assays to determine the levels of the inflammatory cytokines (D) IL-6 (n=5) and (E) TNF-α (n=8) between treatment groups. We used a two-way ANOVA followed by Tukey's post-hoc test to compare treatment groups. Data represent mean ± SEM; we include p-values between 0.1 and 0.01 above each figure, while we have used ** to denote P < 0.01, *** P < 0.001, and **** P < 0.0001.

During the progression of MASLD, persistent liver damage and TGF-β-driven fibrosis prompt macrophage infiltration, promoting tissue inflammation and the development of a proinflammatory precancerous microenvironment [27, 80]. As we observed robust indicators of fibrosis and oxidative stress, we next examined measures of macrophage infiltration and inflammation in the liver. We first stained tissue sections for the macrophage marker F4/80. We observed strong evidence of increased F4/80 staining across the MatExp, PatExp, and DualExp treatment groups in both the Saline and DEN-treated offspring (Fig. 6A-B). We also observed an effect of the DEN treatment, with increased F4/80 staining in DEN offspring compared to Saline-treated males. Using RT-qPCR, we examined the CD86/CD206 ratio in DEN-treated offspring and identified strong evidence of an increased ratio of proinflammatory macrophages across all treatments (Fig. 6C).

Next, we examined the abundance of key proinflammatory cytokines, including IL-6 and TNF-α. Again, we observed strong evidence of an interaction between the MatExp and PatExp treatments, with the abundance of the IL-6 inflammatory cytokine in the DualExp offspring exceeding those induced by either maternal or paternal alcohol use alone (Fig. 6D). We observed this interaction across the Saline and DEN treatments but also noted the effect of the DEN treatment. Emerging preclinical studies suggest that TNF-α may contribute to the progression from MASLD to HCC [81]. We observed modest evidence of increased TNF-α abundance in DualExp offspring in the Saline treatment and strong evidence of increased TNF-α across all treatment groups in DEN-treated offspring, with a DEN-induced increase compared to the Saline treatment (Fig. 6E). Our results indicate that offspring from alcohol-exposed parents exhibit conditions characteristic of a precancerous microenvironment, possibly leading to a more aggressive tumor microenvironment.

Parental alcohol exposures cause increased IL-6 expression across the life course.

**Figure 7.**
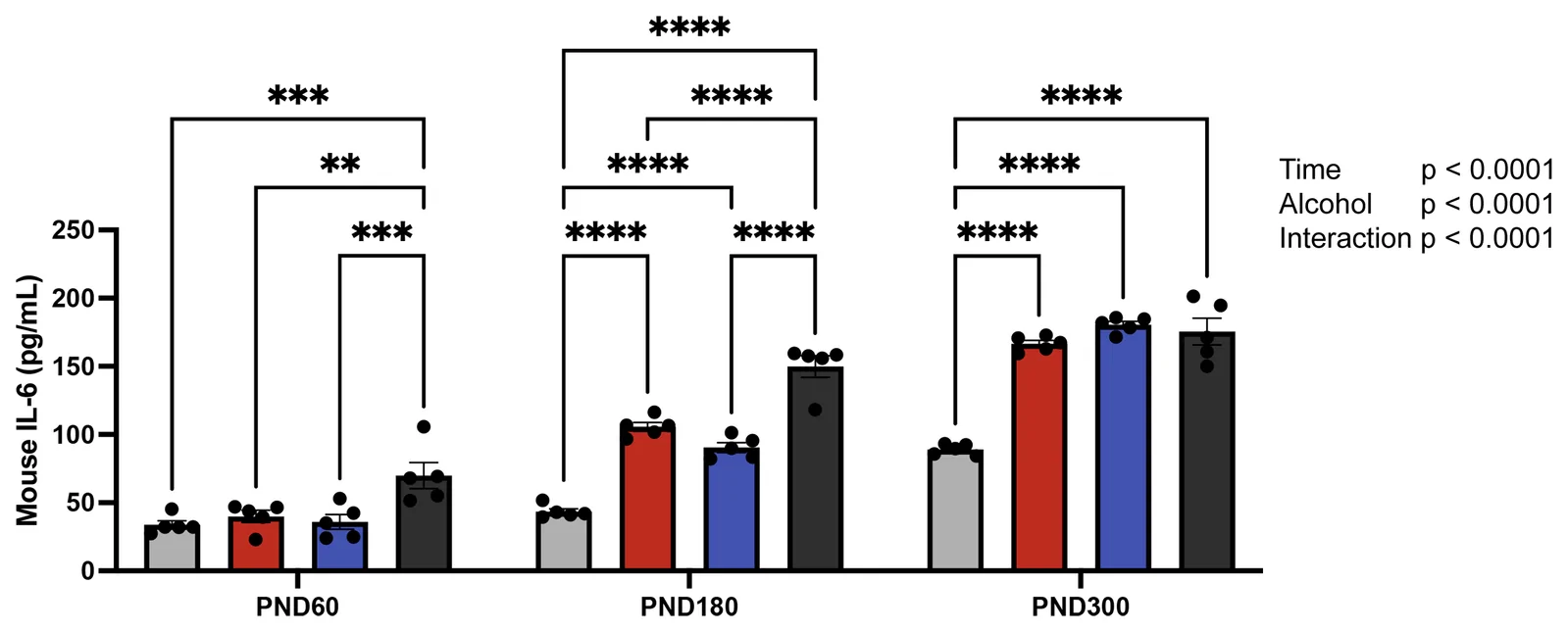
**Dual-parental alcohol exposures promote increased IL-6 production across the life course**. We used an ELISA and a two-way ANOVA to compare IL-6 abundance between treatment groups and across postnatal days (PND) 60, 180, and 300 (n=5). We used Tukey's post-hoc test to compare treatment groups. Data represent mean ± SEM; we have used ** to denote P < 0.01, *** P < 0.001, and **** P < 0.0001.

Consistent with our previous studies examining histological indicators of liver disease, IL-6 abundance also exhibited cumulative increases in the DualExp offspring. In patients with hepatocellular carcinoma, levels of IL-6 are increased and are closely linked to disease occurrence and prognosis [39, 40]. As IL-6/STAT3 signaling is one of the critical signaling pathways involved in HCC initiation and occurrence, and the abundance of this inflammatory cytokine exhibited cumulative effects in DualExp offspring, we examined IL-6 expression across the life course. Using ELISA and a two-way ANOVA, we identified strong evidence of a persistent upregulation of IL-6 in DualExp offspring at all life stages, while, in contrast, MatExp and PatExp offspring only displayed strong evidence of increased IL-6 abundance on postnatal day 180 and beyond (Fig. 7).

## DISCUSSION

The global prevalence of MASLD and HCC is rising, particularly among young men [24]. Although dietary and environmental factors explain some of the increased prevalence, emerging studies indicate that the risk of developing MASLD begins *in utero* and is linked to enduring epigenetic changes in mitochondrial function [28]. As our preclinical work examining the fetal offspring of alcohol-exposed parents consistently identifies the inheritance of altered mitochondrial activity, we hypothesized that preconception paternal and periconceptional maternal alcohol use would increase offspring predisposition to MASLD and the development of hepatocellular carcinoma.

Like parental alcohol exposures, early-life DEN exposures also cause hepatic inflammation, fibrosis, and mitochondrial dysfunction that results in a reduced NAD+/NADH ratio and increased ROS production [44-47]. Here, we used the toxicant DEN as a “second hit” to determine if the offspring of alcohol-exposed parents exhibit increased susceptibility to toxicant-induced liver cancer. We demonstrate that MatExp, PatExp, and DualExp male offspring display enhanced clinical markers of liver damage, including increases in serum AST and ALT, along with increased histological evidence of hepatic fibrosis and steatosis. These adverse changes correlate with increased tumor incidence, multiplicity, and size. Further, the enduring oxidative stress caused by changes in mitochondrial complex I activity reinforces critical pathways promoting cancer progression, including aspects of both profibrotic TGF-β signaling and proinflammatory IL-6 cytokine production. Based on these observations, we theorize that chronic parental alcohol use promotes the epigenetic inheritance of altered baseline mitochondrial function, redox states, and immune function, predisposing the male offspring to a proinflammatory precancerous microenvironment and increased cancer susceptibility.

Our observations complement an existing body of literature examining the impacts of maternal exposure to a high-fat diet, promoting the development of metabolic syndrome and HCC in female offspring, [82, 83] and paternal obesity, which increases offspring susceptibility to breast cancer [84-86]. Consistent with our studies, some of these works also suggest that enhanced tumor susceptibility stems from lasting alterations in the control of cellular bioenergetics. Likewise, rodent models examining other toxicants demonstrate a clear link between paternal preconception stressors and offspring liver dysfunction. For example, paternal exposures to nicotine and environmental toxicants, including tributyltin, also induce increased hepatic steatosis, fibrosis, and altered metabolism in offspring [87, 73, 88]. Our previous studies examining paternal alcohol exposures reveal that both fetal and adolescent offspring exhibit early indicators of MASLD, including increased hepatic fibrosis, steatosis, and alterations in insulin sensitivity [49, 89, 90]. Therefore, multiple paternal dietary stressors and environmental exposures exert heritable epigenetic effects on offspring liver function. These epigenetic changes may interact with adverse lifestyle or nutritional factors, like ultra-processed or fast foods, [91] across generations to compound or reinforce MASLD progression, influencing the heritability of liver disease and hepatocellular carcinoma.

How the memory of parental alcohol exposure persists across the life course to impact offspring liver function is not known. However, our observations of the inflammatory cytokine IL-6 may offer a clue. IL-6 has multiple context-specific functions, both promoting and inhibiting the progression of liver disease [40]. Whereas low-level or controlled IL-6 release drives anti-inflammatory, antioxidant, and pro-growth effects, chronic or high-level IL-6 secretion induces proinflammatory, pro-oxidant, and profibrotic responses [92]. The dualistic actions or hormetic effects of IL-6 likely stem from the ability of this cytokine to influence mitochondrial function [93]. For example, using an inducible humanized IL-6 knock-in mouse model, Nidadavolu and colleagues recently demonstrated that high-level IL-6 stimulation potently disrupts mitochondrial function, affecting multiple nodes in the citric acid cycle and decreasing the cellular production of NADH and ATP [94]. Mitochondrial stress also drives IL-6 production *via* the cGAS-STING pathway [95]. Our transcriptomic analysis identified alterations in the expression of several mitochondrial subunits, including several members of complex I. Notably, early-life DEN treatment also appears to hit these pathways, as our transcriptomic data align with previous examinations of mitochondrial respiration in DEN-induced tumors [44-47].

From these data, we speculate that compromised mitochondrial complex I activity induced by the combined actions of parental alcohol use and early life DEN exposure activates IL-6, which, when combined with increased hepatic oxidative stress and activation of TGF-β signaling, positively reinforces the production of reactive oxygen species and the further deterioration of mitochondrial function. Because the liver is the primary organ responsible for regulating metabolic processes, [79] mitochondria are more abundant in hepatocytes, [96] potentially explaining why this organ shows the greatest sensitivity. Programmed changes in mitochondrial activity, in turn, support tumor cell survival, invasiveness, and progression through alterations in redox homeostasis, metabolism, and cell death signaling pathways [97-99].

In offspring from alcohol-exposed parents, we observed increases in F4/80 staining and increased markers of M1 macrophages, indicative of increased proinflammatory macrophage infiltration. Macrophages are one of the critical immune cells known to produce inflammatory cytokines, including IL-6 and TNF-α, which we observe in MatExp, PatExp, and DualExp offspring. Our findings indicate that offspring from alcohol-exposed parents exhibit an inherent predisposition towards immune dysregulation, leading to a proinflammatory phenotype. Therefore, differences in parental epigenetic programming could contribute to the heterogeneity seen amongst HCC patients, explaining why this disease is so challenging to treat.

Interestingly, at 24 weeks of age, our analysis of IL-6 abundance revealed additive effects in DualExp offspring, aligning with the increased hepatic fibrosis, steatosis, and ALT and AST release, which also exceeded those caused by either maternal or paternal alcohol use alone. Our results suggest that, for some alcohol-related phenotypes, maternal and paternal exposures interact to exacerbate outcomes. Although few studies have examined dual-parental exposures, the existing literature suggests that maternal and paternal stressors interact to intensify adverse outcomes in the next generation [100-104]. As paternal drinking behaviors influence maternal consumption during pregnancy, [105] examining the interactions between maternal and paternal alcohol use will enable a more complete understanding of fetal alcohol spectrum disorder phenotypes and the associated health conditions.

There have been very few clinical studies examining long-term health outcomes in fetal alcohol spectrum disorder patients. The limited epidemiological studies demonstrate that this patient cohort has a decreased life expectancy and increased risk of hospitalizations [106, 107]. Notably, there have been a handful of clinical and preclinical studies suggesting that prenatal ethanol exposure increases the risk of fibrosis and non-alcoholic fatty liver disease [12, 108-110]. However, no studies have determined if parental alcohol use primes the liver microenvironment for the development of liver cancer.

Carcinogenesis can be broken into three stages: 1) tumor initiation, 2) tumor promotion, 3) malignant conversion, and cancer progression. Tumor initiation entails the genetic or epigenetic alterations in a normal cell's DNA, converting it into a cancerous cell, often triggered by factors like carcinogens or genetic predispositions [111]. In contrast, cancer promotion involves the proliferation and expansion of these initiated cells into detectable tumors and is driven by factors like chronic inflammation [111]. Our model demonstrates for the first time that parental alcohol consumption heritably alters mitochondrial activity and ROS production, driving the formation of a proinflammatory environment and very likely contributing to tumor promotion.

Although the data we present is compelling, this work has some limitations. First, although we can consistently identify lasting changes in mitochondrial activity and immune function induced by parental alcohol exposure, the transcriptional changes we observe are modest, and we have yet to identify epigenetic changes in the offspring that could explain these differences. Therefore, how this memory transmits from parent to offspring, persists across the life course, and impacts disease susceptibility remains unclear. It is possible that the phenomena we describe in our model are a form of maladaptive mitochondrial adaptation. Mitochondrial adaptation refers to the processes by which mitochondria adjust their structure, function, and metabolism in response to cellular and environmental cues [112]. For example, exercise, cold exposure, and caloric restriction induce beneficial adaptations in mitochondrial function that are vital for maintaining cellular and organismal health. In contrast, the ischemic and reperfusion events during stroke promote a lasting deficit in mitochondrial activity, also specifically impacting complex I, that persists long after the initial event [113]. Similarly, chronic alcohol exposure impedes mitochondrial complex I and promotes a lasting reduction in the hepatic NAD+/NADH ratio [114, 115]. We speculate that the alcohol-induced changes in sperm noncoding RNAs observed by us and others adversely impact offspring mitochondrial function during early preimplantation development and that, due to the plastic nature of embryonic bioenergetics, these deficits persist through development into adult life. In support of this assertion, studies examining the transient induction of oxidative stress via inhibition of superoxide dismutase 2 (SOD2) during early development impart lasting impacts on adult liver function [116]. However, we cannot definitively say whether alterations in mitochondrial activity or immune function drive disease predisposition or are merely additional symptoms. Further studies examining early developmental stages will help disentangle the mechanism of heritability. Finally, because we focused our analysis on male offspring, we do not know if parental alcohol use imparts female susceptibility to DEN-induced HCC, as observed in studies examining gestational exposure to a high-fat diet [83].

Alcohol consumption is a significant factor contributing to hepatocellular carcinoma. For the first time, we demonstrate that HCC development and susceptibility in the offspring of alcohol-exposed parents can arise through presumptive epigenetic mechanisms instead of direct alcohol exposure. If our preclinical studies translate to humans, individuals with fetal alcohol spectrum disorder may inherit a heightened risk for diminished mitochondrial activity, increased inflammation, and the progression of cancer. This increased disease susceptibility emphasizes the vulnerability of this population and highlights the need to (1) expand public health messaging to include men, (2) caution couples on the potential cumulative dangers of dual parental alcohol use, and (3) do more epidemiological studies on fetal alcohol spectrum disorder patients studying the long-term health effects.

## Supplementary Materials

The Supplementary data can be found online at: www.aginganddisease.org/EN/10.14336/AD.2024.1372.

## RNA-seq Data

Transcriptomic data are available under GEO Accession GSE273675. No other large datasets were created or analyzed in this study.
