# Supplementary material for: Parental Alcohol Use Disrupts Offspring Mitochondrial Activity, Promoting Susceptibility to Toxicant-Induced Liver Cancer
Source: Aging Dis. 2025 Jan 25;17(1):383–404. doi: 10.14336/AD.2024.1372 (PMC12727083; doi:10.14336/AD.2024.1372)
Supplement: Supplementary file 1 — The Supplementary data can be found online at: www.aginganddisease.org/EN/10.14336/AD.2024.1372. [file AD-17-1-383-s.pdf]

SUPPLEMENTARY DATA

Parental Alcohol Use Disrupts Offspring Mitochondrial Activity, Promoting Susceptibility to Toxicant-Induced Liver Cancer

Alison Basel, Sanat S. Bhadsavle, Katherine Z. Scaturro, Grace K. Parkey, Yava Jones-Hall, Michael C. Golding

© 2024. Basel A. et al. Published online at http://www.aginganddisease.org/EN/10.14336/AD.2024.1372

SUPPLEMENTARY DATA

Supplementary Table 1. Detailed descriptions of each statistical test and sample size for each Figure.

**Litter and sex information per treatment group**			
**Treatment**	**Number of Litters**	**Number of Males**	**Number of Females**
Saline Control	3	9	12
Saline Maternal	3	9	13
Saline Paternal	4	12	11
Saline Dual	3	13	10
DEN Control	4	11	10
DEN Maternal	4	13	11
DEN Paternal	3	9	14
DEN Dual	5	10	16
**Graph**	**Statistical Test**	**Sample Size**	**Outliers**
Figure 1: A multiplex mouse model to determine the impacts of parental alcohol use on offspring predisposition to toxicant-induced hepatocellular carcinoma.			
**C-D:** Sire fluid consumption and body weight	Two-way ANOVA, multiple comparisons using Sidak.	n = 9 control<br>9 ethanol	0
**E:** Average daily dose	Ordinary One-way ANOVA, multiple comparisons using Tukeys.	n = 17 paternal<br>18 maternal preconception<br>18 maternal gestation	1 maternal preconception
**F:** Paternal daily dose	Unpaired t test.	n = 8 paternal<br>9 dual	0
**G-H:** Maternal fluid consumption	Two-way ANOVA, multiple comparisons using Sidak.	n = 14 preconception control<br>12 preconception ethanol<br>18 gestation control<br>18 gestation ethanol	1 pre-conception control<br>2 gestation control
**I:** Maternal daily dose	Two-way ANOVA, multiple comparisons using Sidak.	n = 8 maternal<br>10 dual	1 dual preconception
**J:** Maternal daily food consumption	Two-way ANOVA, multiple comparisons using Sidak.	n = 14 preconception control<br>12 preconception ethanol<br>18 gestation control<br>18 gestation ethanol	1 pre-conception control<br>1 gestation control
**K-L:** Gestational caloric intake and weight gain	Unpaired t test.	K: n = 12 control<br>18 ethanol<br>L: n = 14 control<br>18 ethanol	0
**M-N:** Gestation length and litter size	Kruskal-Wallis, multiple comparisons using Dunn's.	M: n = 7 control<br>8 maternal<br>7 paternal<br>10 dual<br>N: n = 7 control<br>8 maternal<br>7 paternal<br>10 dual	L: 1 control
**O:** Sex ratio	Chi-Square analysis followed by Fisher's Exact	n = 42 control<br>46 maternal<br>46 paternal	0

© 2024. Basel A. et al. Published online at http://www.aginganddisease.org/EN/10.14336/AD.2024.1372

		test for individual comparisons.	49 dual	
Figure 2: Parental alcohol use increases tumor incidence, number, and diameter in a mouse model of DEN-induced liver injury.				
**A-D:**	Offspring weekly body weight and food consumption	Two-way ANOVA, multiple comparisons using Tukey.	A-B: n = 9 control<br>9 maternal<br>12 paternal<br>13 dual<br>C-D: n = 11 control<br>13 maternal<br>9 paternal<br>10 dual	0
**E:**	Liver to body weight	We inserted organ weights into Excel, then divided by total body weight. Two-way ANOVA, multiple comparisons using Tukey.	Saline: n = 9 control<br>9 maternal<br>12 paternal<br>13 dual<br>DEN: n = 11 control<br>13 maternal<br>9 paternal<br>10 dual	1 saline maternal<br>1 saline paternal<br>1 saline dual
**H-I:**	Animals with tumors and number of tumors	Chi-Square analysis followed by Fisher's Exact test for individual comparisons.	n = 9 control<br>9 maternal<br>12 paternal<br>13 dual	0
**J:**	Largest tumor diameter	Kruskal-Wallis, multiple comparisons using Dunn's.	n = 5 control<br>5 maternal<br>5 paternal<br>6 dual	0
**K:**	PCNA quantification	Ordinary One-way ANOVA, multiple comparisons using Tukeys.	n = 7 control<br>5 maternal.<br>8 paternal<br>6 dual	0
Figure 3: Maternal and paternal alcohol use interact in driving the progression of toxicant-induced liver disease.				
**B & D:**	Histology quantification	Two-way ANOVA, multiple comparisons using Tukeys.	Saline: n = 8 control<br>8 maternal<br>8 paternal<br>8 dual<br>DEN: n = 8 control<br>8 maternal<br>8 paternal<br>8 dual	0
**E-F:**	ALT and AST quantification	Two-way ANOVA, multiple comparisons using Fisher's LSD.	Saline: n = 8 control<br>8 maternal<br>8 paternal<br>8 dual<br>DEN: n = 8 control<br>8 maternal<br>8 paternal<br>8 dual	E: 1 saline control<br>1 DEN paternal<br>F: 1 saline maternal
**H-I:**	Blinded histology scores	Two-way ANOVA, multiple comparisons using Fisher's LSD.	Saline: n = 8 control<br>8 maternal<br>8 paternal<br>8 dual<br>DEN: n = 8 control<br>8 maternal<br>8 paternal<br>8 dual	0
Figure 4: Transcriptomic analysis reveals parental alcohol exposures disrupt mitochondrial function and Transforming Growth Factor beta (TGF-B) signaling.				
**A:**			Saline: n = 6 control	0

© 2024. Basel A. et al. Published online at http://www.aginganddisease.org/EN/10.14336/AD.2024.1372

	CYP2E1 quantification	Two-way ANOVA, multiple comparisons using Tukeys.	DEN: n =	6 maternal<br>6 paternal<br>6 dual<br>6 control<br>6 maternal<br>6 paternal<br>6 dual	
**F-I:**	RT-qPCR analysis	Ordinary One-way ANOVA, multiple comparisons using Tukeys.	n =	8 control<br>8 maternal<br>8 paternal<br>8 dual	0
Figure 5: Parental alcohol exposures cause persistent mitochondrial dysfunction, increased mitochondrial stress, and transcriptional suppression of the antioxidant response in offspring liver.					
**A:**	NAD/NADH Assay	Ordinary One-way ANOVA, multiple comparisons using Tukeys	n =	4 control<br>4 maternal<br>4 paternal<br>4 dual	0
**B-D**	Mitochondrial complex 1 and malate dehydrogenase activities	Repeated measures ANOVA, multiple comparisons using Tukeys	n =	8 control<br>8 maternal<br>8 paternal<br>8 dual	0
**E-F:**	ROS quantification	Ordinary One-way ANOVA, multiple comparisons using Tukeys	n =	6 control<br>6 maternal<br>6 paternal<br>6 dual	0
**G-L:**	RT-qPCR analysis	Ordinary One-way ANOVA, multiple comparisons using Tukeys	n =	8 control<br>8 maternal<br>8 paternal<br>8 dual	Nrf2: 1 paternal<br>Fgf21: 1 paternal
Figure 6: Parental alcohol exposures program immune dysfunction, supporting development of an inflammatory precancerous microenvironment.					
**B:**	Immuno-histochemistry quantification	Two-way ANOVA, multiple comparisons using Tukeys.	Saline: n =<br>DEN: n =	4 control<br>4 maternal<br>4 paternal<br>4 dual<br>4 control<br>4 maternal<br>4 paternal<br>4 dual	0
**C:**	RT-qPCR analysis	Ordinary One-way ANOVA, multiple comparisons using Tukeys	n =	8 control<br>8 maternal<br>8 paternal<br>8 dual	2 control<br>1 dual
**D:**	IL-6 quantification	Two-way ANOVA, multiple comparisons using Tukeys.	Saline: n =<br>DEN: n =	5 control<br>5 maternal<br>5 paternal<br>5 dual<br>5 control<br>5 maternal<br>5 paternal<br>5 dual	0
**E:**	TNF-α	Two-way ANOVA, multiple comparisons using Fisher's LSD.	Saline: n =<br>DEN: n =	8 control<br>8 maternal<br>8 paternal<br>8 dual<br>8 control<br>8 maternal<br>8 paternal<br>8 dual	0
Figure 7: Dual-parental alcohol exposures promote increased IL-6 production across the life course.					
			PND60 n =	5 control	0

© 2024. Basel A. et al. Published online at http://www.aginganddisease.org/EN/10.14336/AD.2024.1372

SUPPLEMENTARY DATA

			5 maternal<br>5 paternal<br>5 dual
IL-6 production across time	Two-way ANOVA, multiple comparisons using Tukeys.	PND180 n =	5 control<br>5 maternal<br>5 paternal<br>5 dual
		PND300 n=	5 control<br>5 maternal<br>5 paternal<br>5 dual

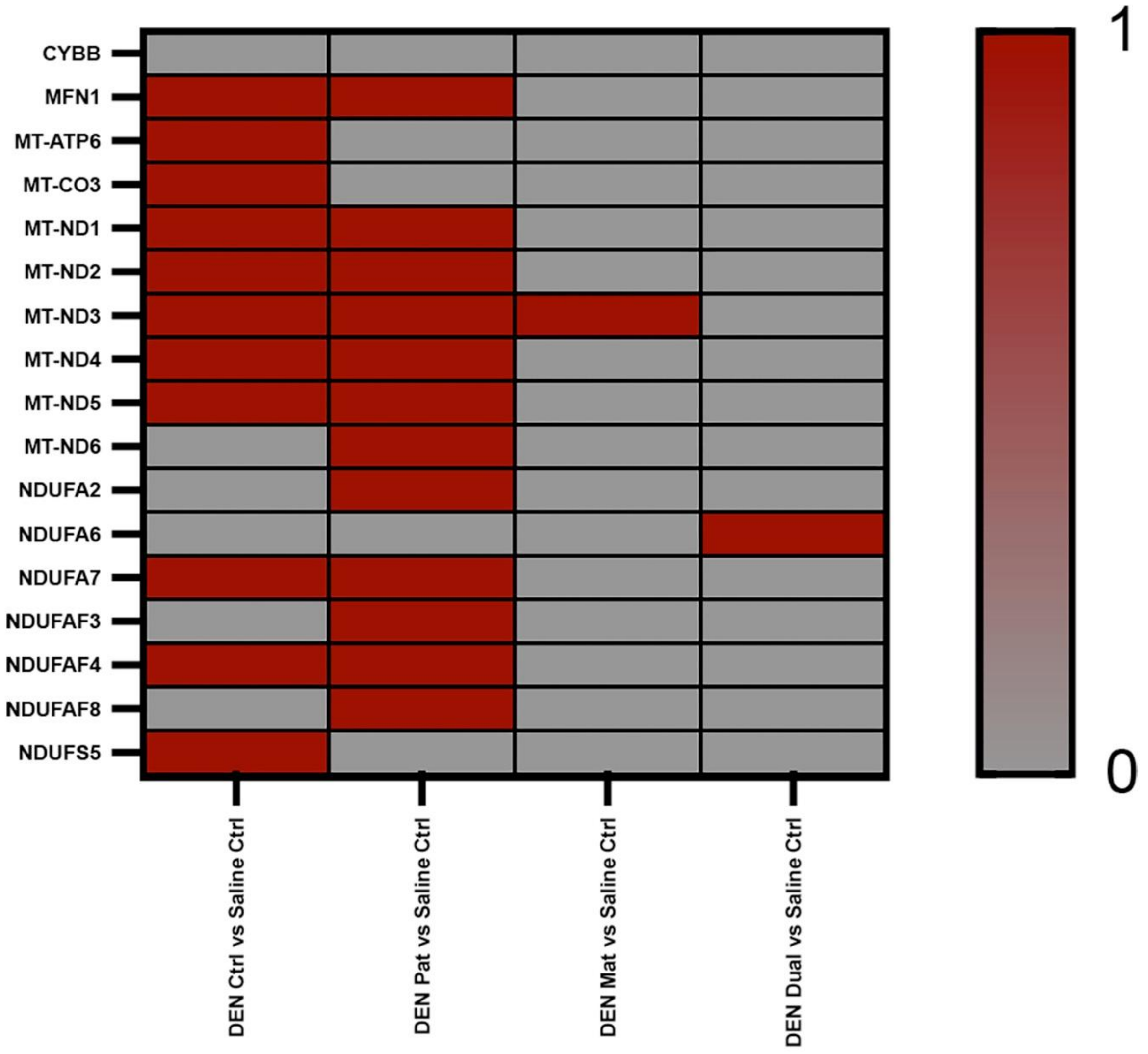

Supplementary Figure 1. Heatmap comparing the number of differentially expressed genes regulating mitochondrial function identified during analysis of the RNA-sequencing datasets.

© 2024. Basel A. et al. Published online at http://www.aginganddisease.org/EN/10.14336/AD.2024.1372
